# Salt Dependence of DNA Binding Activity of Human Transcription Factor Dlx3

**DOI:** 10.3390/ijms23169497

**Published:** 2022-08-22

**Authors:** Ho-Seong Jin, Juyeon Son, Yeo-Jin Seo, Seo-Ree Choi, Hye-Bin Ahn, Youyeon Go, Juhee Lim, Kwang-Im Oh, Kyoung-Seok Ryu, Joon-Hwa Lee

**Affiliations:** 1 Department of Chemistry and RINS, Gyeongsang National University, Jinju 52828, Korea; 2 Protein Structure Research Team, Korea Basic Science Institute, Ochang 28119, Korea

**Keywords:** transcription factor, NMR, DNA–protein interaction, salt dependence, base-pair stability, chemical shift perturbation

## Abstract

Distal-less 3 (Dlx3) is a homeobox-containing transcription factor and plays a crucial role in the development and differentiation process. Human Dlx3 consists of two transactivation domains and a homeobox domain (HD) that selectively binds to the consensus site (5′-TAATT-3′) of the DNA duplex. Here, we performed chemical shift perturbation experiments on Dlx3-HD in a complex with a 10-base-paired (10-bp) DNA duplex under various salt conditions. We also acquired the imino proton spectra of the 10-bp DNA to monitor the changes in base-pair stabilities during titration with Dlx3-HD. Our study demonstrates that Dlx3-HD selectively recognizes its consensus DNA sequences through the α3 helix and L1 loop regions with a unique dynamic feature. The dynamic properties of the binding of Dlx3-HD to its consensus DNA sequence can be modulated by varying the salt concentrations. Our study suggested that this unique structural and dynamic feature of Dlx3-HD plays an important role in target DNA recognition, which might be associated with tricho-dento-osseous syndrome.

## 1. Introduction

The distal-less transcription factor was originally discovered in *Drosophila melanogaster* and was found to affect the legs and antennae of the fly [1,2]. In mammals, the distal-less family of transcription regulators includes six members and clusters along different chromosomes [2,3,4]. Among them, distal-less 3 (Dlx3) is a homeobox-containing transcription factor and has a crucial function in the development and differentiation process [2,5,6]. In vertebrates, Dlx3 is expressed in the teeth, hair follicles, and skin, and has crucial functions in tooth development, hair differentiation, and bone formation [7,8,9,10,11].

**Figure 1:**
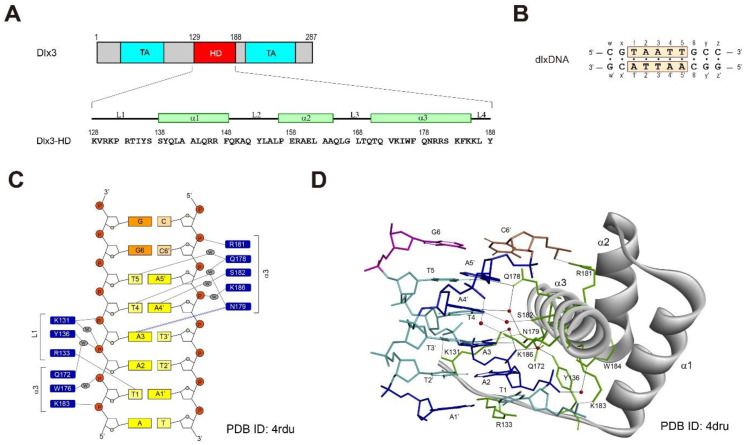
(**A**) Domain structure of Dlx3 and sequence contexts of Dlx3-HD. Numbering and secondary structure elements for Dlx3-HD are shown on top of the sequence. HD and TA indicate homeobox and transactivation domains, respectively. (**B**) Sequence contexts of the dlxDNA duplex. (**C**) Residues of Dlx3-HD involved in intermolecular interaction with DNA reported in previous studies. (**D**) The crystal structure of the Dlx5–DNA complex [21] illustrates intermolecular hydrogen bonding interactions. The red spheres indicate the water molecules involved in water-mediated intermolecular H-bond interactions. The black solid lines indicate the intermolecular H-bond interaction.

The human Dlx3 transcription factor consists of a central homeobox domain (HD) and two transactivation (TA) domains, which are located on either side of the HD region (Figure 1A) [12]. The transcription activity of Dlx3 depends on these two TA domains [12,13]. Mutations of Dlx3 cause tricho-dento-osseous (TDO) syndrome, a genetic disorder manifested by taurodontism, hair abnormalities, and increased bone density in the cranium [14,15,16]. It was first reported that a four base-pair deletion in the *DLX3* gene was associated with TDO [14]. The appendicular skeleton in TDO patients indicates that the Dlx3 function is important in both intramembranous and endochondral bone formation [16]. To date, six different mutations in the *DLX3* gene have been identified in TDO patients of various races including Irish-American, Finnish, and Chinese [17,18,19].

The optimal DNA binding site of Dlx3 comprises a 5′-TAATT-3′ motif, which was determined by PCR-based methods and mobility shift competition assays [12]. This consensus sequence of the DNA binding site of Dlx3 was also confirmed by the consecutive affinity-purification systematic evolution of ligands by exponential enrichment (CAP-SELEX) [20]. The flanking bases of the TAATT core are degenerate, but Dlx3 has a preference for 5′-G-(A/C)-TAATT-(A/G)-(G/C)-3′ [12].

The crystal structural study of the HD of distal-less 5 (Dlx5-HD) complexed with a DNA duplex revealed that the α3 helix directly contacts DNA bases through the major groove of the DNA (Figure 1C,D) [21]. The side-chain of N187 (N179 in Dlx3) exhibits two hydrogen-bonding (H-bonding) interactions with the A3 base of the consensus 5′-T1-A2-A3-T4-T5-3′ site (Figure 1C) [21]. Q186 (Q178 in Dlx3) recognizes the T5 base via water-mediated H-bonding interactions (Figure 1C) [21]. In addition, R141 (R133 in Dlx3) in the L1 loop also shows a H-bonding interaction with the T1 base (Figure 1C) [21]. An NMR study of the Dlx5–DNA complex revealed that Dlx5-HD forms a complex in solution with DNA and shows an exchange between free and complex forms with an intermediate NMR timescale [21]. It has been reported that the substitution of Q178, which is highly conserved during evolution, by Arg causes TDO syndrome [6,19]. R133P, I175S, and S182F are also causative mutations for TDO [18,22]. This means that the Dlx3-HD plays an important role in the biological function of Dlx3, which is linked to TDO.

For the site-specific DNA binding proteins, the target search rate depends on DNA length as well as salt concentration [23]. For example, for the DNA restriction enzyme, LacI, the search rate for short DNA is independent of salt concentration, but it shows a peak pattern for longer DNA as salt concentration is increased [23]. It has been reported that DNA binding proteins show an uncoupled translation–rotation transition at higher salt concentrations, but this is less dominant at low salt concentrations [24].

In order to understand the molecular mechanism of the DNA binding process of the homeobox domain of Dlx3 (Dlx3-HD), we performed NMR experiments on Dlx3-HD complexed with a 10-base-paired (10-bp) DNA duplex containing the consensus sequence, d(CGTAATTGCC)/d(GGCAATTACG) (Figure 1B, labelled dlxDNA). We carried out chemical shift perturbation (CSP) experiments on the Dlx3-HD–dlxDNA complex under various NaCl concentrations to find the correlation between the DNA binding of Dlx3-HD and salt concentration. We also acquired the imino proton spectra of dlxDNA to monitor the change in the base-pair stability during titration with Dlx3-HD. This study yields valuable insights into the molecular mechanism of the DNA recognition mode of Dlx3 transcription factors.

## 2. Results

### 2.1. Assignments of Imino Proton Resonances of dlxDNA

**Figure 2:**
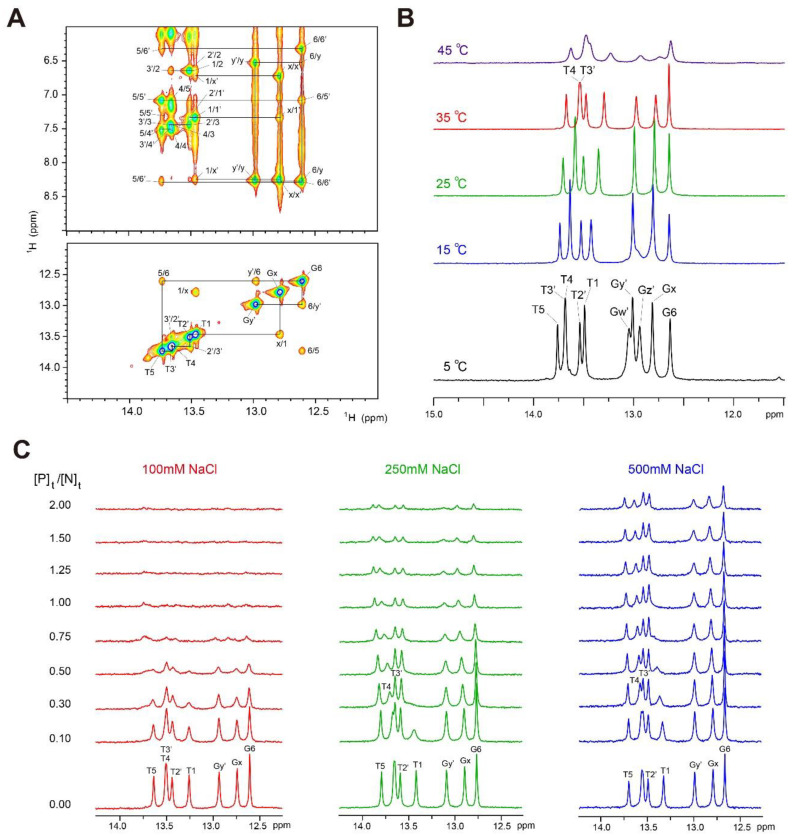
(**A**) Gradient-11-echo NOESY spectra (mixing time = 250 ms) of dlxDNA at 5 °C in 90% H_2_O/10% D_2_O NMR buffer containing 10 mM sodium phosphate (pH = 6.0) and 100 mM NaCl. Solid lines indicate (upper) NOE cross-peaks between imino protons and their own and neighboring H2 or amino protons and (lower) imino-imino connectivities. (**B**) Temperature-dependent 1D imino proton spectra of dlxDNA in NMR buffer. (**C**) The 1D imino proton spectra of dlxDNA at 35 °C upon titration with Dlx3-HD in NMR buffer containing 100 (left), 250 (middle), and 500 mM NaCl (right).

All imino proton resonances from the Watson–Crick base-pairs of dlxDNA were assigned by the strong G-imino to C-amino or T-imino to A-H2 NOE cross-peaks in a two-dimensional (2D) gradient-11-echo NOESY (mixing time: 250 ms) at 5 °C, as shown in Figure 2A. Figure 2B shows the temperature dependence of the imino proton spectra of the dlxDNA. All imino protons except the terminal Gw’ and Gz’ showed sharp resonances up to 35 °C (Figure 2B). After that, the imino proton resonances became severely broadened when the temperature was 45 °C (Figure 2B). These results may demonstrate that the dlxDNA forms a stable double helix up to 35 °C.

### 2.2. Change in 1D Imino Proton Spectra of dlxDNA in Complex with Dlx3-HD

Figure 2C shows the changes in the 1D imino proton spectra of dlxDNA upon titration with Dlx3-HD at 35 °C under various [NaCl]. At [NaCl] = 100 mM, as the protein-to-DNA molar ([P]_t_/[N]_t_) ratio is increased, all imino resonances become severely line-broadened until they completely disappear at a [P]_t_/[N]_t_ ratio > 1.0 (Figure 2C). These results demonstrate that the base-pairs of dlxDNA are significantly destabilized upon binding to Dlx3-HD at [NaCl] = 100 mM.

At [NaCl] = 250 mM, when the [P]_t_/[N]_t_ ratio is > 1.0, most imino proton resonances became line-broadened, but they can still be observed at a [P]_t_/[N]_t_ ratio = 2.0, in contrast to those at [NaCl] = 100 mM (Figure 2C). Interestingly, the T1 imino resonance completely disappeared only at a [P]_t_/[N]_t_ ratio = 0.3 at [NaCl] = 250 mM (Figure 2C). These data indicate that the T1·A1’ base-pair is more severely destabilized than other base-pairs upon binding to Dlx3-HD under high [NaCl] conditions. In addition, the T4 imino resonance showed a significant down-filed shift with line-broadening as the [P]_t_/[N]_t_ ratio increased (Figure 2C), indicating a conformation change and the thermodynamic instability of the T4·A4’ base-pair. The crystal structure of the Dlx5–DNA complex showed that the R133 (R141 of Dlx5) side-chain exhibited a H-bonding interaction with the carbonyl group on the T1 base of dlxDNA (Figure 1D) [21]. This study also found that the T4 base of DNA is recognized by the water-mediated H-bonding interaction of Q178 and N179 (Figure 1D) [21]. Thus, our results indicate that the intermolecular H-bonding interactions of R133 and Q178/N179 play important roles in the stabilization of the T1·A1’ and T4·A4’ base-pairs, respectively, and that these are very sensitive to the ionic strength of the solution.

At [NaCl] = 500 mM, the Dlx3-HD caused significant perturbation results, but its efficiency is less than that of [NaCl] = 250 mM (Figure 2C). For example, T1 resonance was still observed up to [P]_t_/[N]_t_ = 0.75 at [NaCl] = 500 mM, whereas it completely disappeared at [P]_t_/[N]_t_ = 0.3 at [NaCl] = 250 mM (Figure 2C). A similar effect was also observed for the T4 resonance (Figure 2C).

### 2.3. ^1^H/^15^N-HSQC Spectra of Dlx3-HD at Various [NaCl]

**Figure 3:**
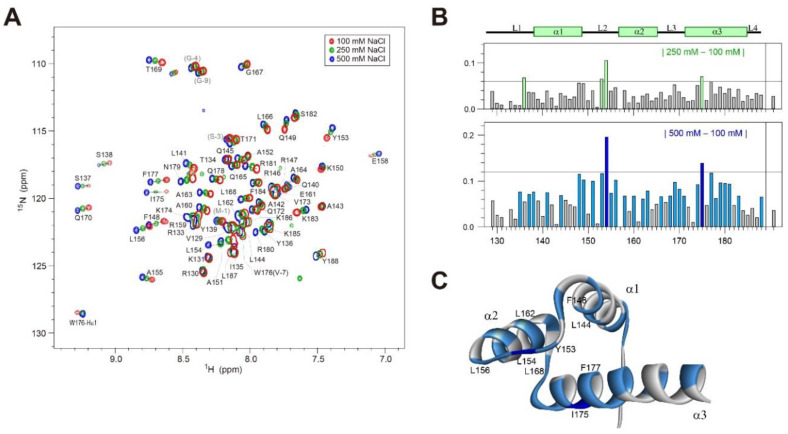
(**A**) Superposition of ^1^H/^15^N-HSQC spectra of free Dlx3-HD at 100 mM (red), 250 mM (green), or 500 mM NaCl (blue) at 35 °C. (**B**) The average chemical shift differences (Δδ_avg_) between [NaCl] of 100 and 250 mM (upper) and of 100 and 500 mM (lower) for free Dlx3-HD at 35 °C. (**C**) Mapping the location of the residues with a large Δδ_avg_ between [NaCl] of 100 and 500 mM onto the structure of free Dlx3-HD. The colors used to illustrate the Δδ_avg_ are blue (>0.12 ppm) and light blue (0.06–0.12 ppm).

Resonance assignments of Dlx3-HD were made by analyzing their 3D NOESY-^1^H/^15^N-HSQC and TOCSY-^1^H/^15^N-HSQC spectra. A superposition of the ^1^H/^15^N-HSQC spectra for Dlx3-HD at 35 °C under various NaCl concentrations is shown in Figure 3A. As [NaCl] is increased from 100 to 250 mM, all amide resonances slightly move, with the average chemical shift changes (Δδ_avg_) being in the range 0.01–0.11 (Figure 3B). When [NaCl] = 500 mM, the Δδ_avg_ values become much larger compared to those of the 250 mM NaCl condition (Figure 3B). For example, the L154 (in the L2 loop) and I175 (in the α3 helix) amide protons showed Δδ_avg_ values larger than 0.12 ppm (Figure 3B). In addition, significant chemical shift changes were observed in other residues in the L2 and α3 regions (Figure 3B). The amide residues, which exhibited significant Δδ_avg_ values upon an increment in salt concentration, are shown in Figure 3C.

### 2.4. ^1^H/^15^N-HSQC Spectra of Dlx3-HD–dlx3DNA Complex at 100 mM NaCl

**Figure 4:**
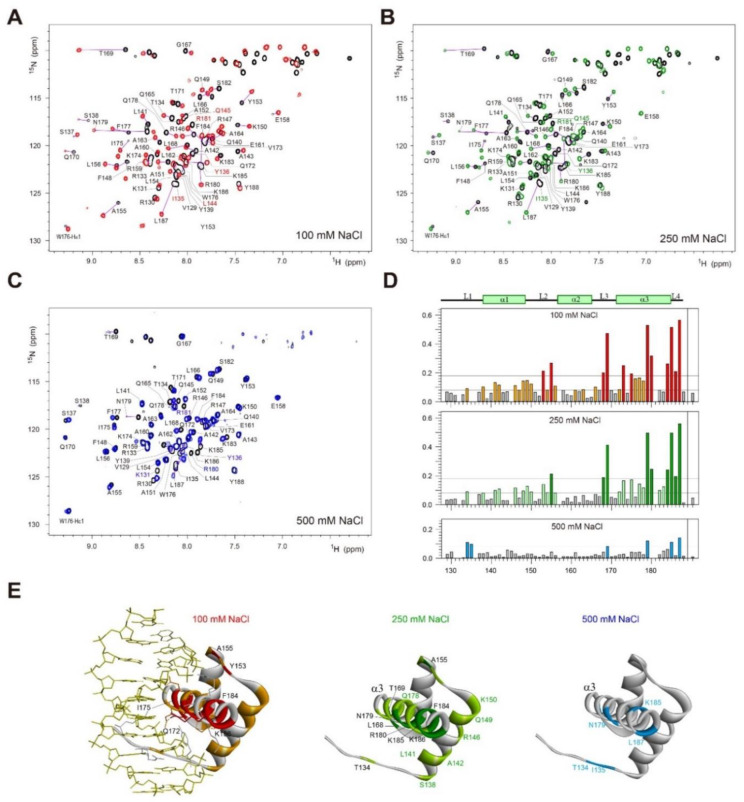
Superposition of ^1^H/^15^N-HSQC spectra of free Dlx3-HD (black) and Dlx3-HD–dlxDNA complex ([N]_t_/[P]_t_ = 1.5, red, green, or blue) at (**A**) 100 mM, (**B**) 250 mM, or (**C**) 500 mM NaCl at 35 °C. (**D**) The average chemical shift changes (Δδ_avg_) in Dlx3-HD upon binding to dlxDNA at 100 mM (upper), 250 mM (middle), or 500 mM NaCl (lower) at 35 °C. (**E**) Mapping the location of the residues with a large Δδ_avg_ onto the structure of the Dlx3-HD–dlxDNA complex. The colors used to illustrate the Δδ_avg_ are red, green, or blue (>0.18 ppm) and orange, pale green, or light blue (0.08–0.18 ppm) (the same color coding is used in panel (**D**)).

A superimposition of the ^1^H/^15^N-HSQC spectra of free Dlx3-HD and the Dlx3-HD–dlxDNA complex ([N]_t_/[P]_t_ = 1.5) at 100 mM NaCl is shown in Figure 4A. The weighted average ^1^H/^15^N backbone chemical shift differences (Δδavg) between the free and complex forms were determined for each residue using Equation (1) (Figure 4D). Many residues in the L3-α3-L4 regions showed significant chemical shift changes larger than 0.18 ppm (Figure 4D). These results indicate a direct interaction between their side chains and dlxDNA, as was reported in the crystal structure of the Dlx5–DNA complex (Figure 1D) [21]. In addition, significant chemical shift changes were observed in the α1 helix (Figure 4D). Surprisingly, most residues in the L1 loop exhibited Δδavg values of less than 0.12 ppm (Figure 4D), although the side-chains of K131, R133, and Y136 formed H-bonding interactions with the phosphate backbone of DNA (Figure 1) [21]. Instead, the amide signals of I135 and Y136 in the L1 loop and L144 and Q145 in the α1 helix disappeared upon binding to the DNA substrate (Figure 4A).

Interestingly, residues Y153 and A155 in the L2 loop also have Δδavg values > 0.18 ppm (Figure 4D), although it was reported that this region did not show any intermolecular interaction with dlxDNA [21].

### 2.5. ^1^H/^15^N-HSQC Spectra of Dlx3-HD–dlx3DNA Complex at 250 and 500 mM NaCl

Superimpositions of the ^1^H/^15^N-HSQC spectra of free Dlx3-HD and the Dlx3-HD–dlxDNA complex ([N]_t_/[P]_t_ = 1.5) at 250 and 500 mM NaCl are shown in Figure 4B,C, respectively. Although [NaCl] increased from 100 to 250 mM, significant chemical shift changes were observed for residues in the α1 and L3-α3-L4 regions (Figure 4D,E). Similar to 100 mM NaCl, the amide signals of I135, Y136, L144, and Q145 disappeared in the Dlx3-HD–DNA complex at 250 mM (Figure 4B). These data indicated that the DNA binding affinity of Dlx3-HD was little affected by change in [NaCl] up to 250 mM.

The magnitude of each chemical shift change at 500 mM NaCl was reduced compared with that measured at 100 and 250 mM NaCl (Figure 4D). For example, the N179 amide signal was moved with Δδavg = 0.121 ppm by dlxDNA addition at 500 mM, whereas this residue had the Δδavg values of 0.529 and 0.497 ppm at 100 and 250 mM NaCl, respectively (Figure 4D). Similarly, all amide signals in the L3-α3-L4 region were around five times less perturbed at 500 mM than at 100 and 250 mM NaCl (Figure 4D). In addition, residues in the α1 helix and L2 loop exhibited few chemical shift changes at 500 mM NaCl in contrast to those of 100 and 250 mM NaCl (Figure 4D). This implies that the DNA binding affinity is much weaker at 500 mM NaCl than at lower salt concentrations. The most surprising feature is that residues in the L1 loop showed a salt concentration-independent chemical shift perturbation pattern (Figure 4D). For example, the Δδavg value of T134 is 0.091, 0.091, and 0.109 ppm at 100, 250, and 500 mM NaCl, respectively (Figure 4D).

### 2.6. Change in ^1^H/^15^N-HSQC Spectra of Dlx3-HD in Complex with dlxDNA

**Figure 5:**
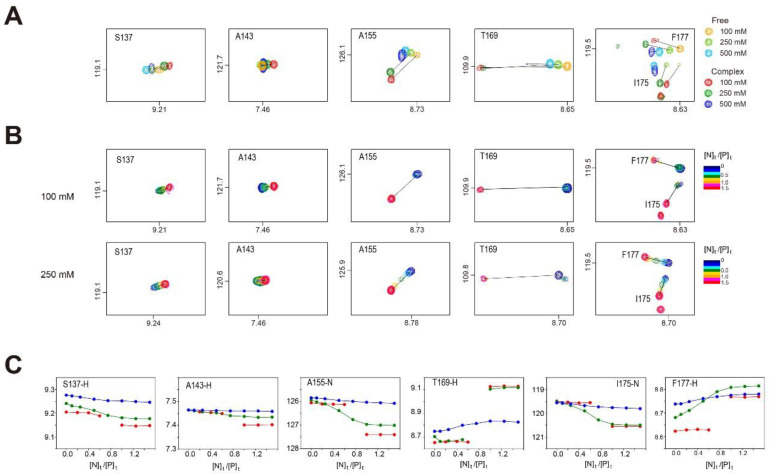
(**A**) Comparison of ^1^H/^15^N-HSQC peaks of S137, A143, A155, T169, I175, and F177 in free Dlx3-HD and Dlx3-HD–dlxDNA complex ([N]_t_/[P]_t_ = 1.5) at 100, 250, or 500 mM NaCl at 35 °C. (**B**) Comparison of ^1^H/^15^N-HSQC peaks of S137, A143, A155, T169, I175, and F177 of Dlx3-HD at 100 mM (upper) or 250 mM NaCl (lower) at 35 °C. The cross-peak color changes gradually from dark blue (free) to red (complex) according to the [N]_t_/[P]_t_ ratio. (**C**) Chemical shift values of S137-H, A143-H, A155-N, T169-H, I175-N, and F177-H signals of Dlx3-HD upon titration with dlxDNA as a function of the [N]_t_/[P]_t_ ratio at 100 (red), 250 (green), or 500 mM NaCl (blue) at 35 °C.

To further clarify the salt dependence of the chemical shift perturbation, the ^1^H/^15^N-HSQC spectra were acquired as a function of the [N]_t_/[P]_t_ ratio under various [NaCl] conditions. Most amide signals for Dlx3-HD were strong at each titration point at 250 mM NaCl (Figure 5), indicating that DNA binding at 250 mM NaCl is a fast exchange process. However, the amide signals exhibited slow exchange at 100 mM NaCl on the NMR time scale (Figure 5). The F177 amide signal in the α3 helix showed significant movement at 100 and 250 mM NaCl compared to 500 mM NaCl (Figure 5A). At 100 mM NaCl, this F177 resonance was located at δ_H_ = ~8.6 ppm and δ_N_ = ~119.5 ppm at [N]_t_/[P]_t_ ≤ 0.5 (Figure 5B). However, this resonance immediately moved to δ_H_ = ~8.77 and δ_N_ = ~118.2 ppm, when the [N]_t_/[P]_t_ ratio became greater than 1.0 (Figure 5B). At 250 mM NaCl, the F177 resonance at δH = 8.68 and δN = 118.7 ppm continuously moved to δ_H_ = 8.81 and δ_N_ = 118.4 ppm, as the [N]_t_/[P]_t_ ratio increased (Figure 5B). Similar results were observed for the S137, A143, A155, T169, and I175 resonances (Figure 5B). The chemical shift changes of these resonances are plotted as a function of the [N]_t_/[P]_t_ ratio in Figure 5C. These results indicate that when [NaCl] increases from 100 to 250 mM, the DNA binding process of Dlx3-HD becomes more dynamic, despite its binding affinity not being affected.

When [NaCl] = 500 mM while maintaining a fast exchange of the amide signals on the NMR time scale, the degree of CSP became much smaller compared to 250 mM NaCl (Figure 5C).

## 3. Discussion

TDO syndrome has been linked to a frameshift mutation (GGGG deletion in *DLX3* gene) just downstream of the Dlx3-HD [14,15]. Interestingly, the missense-type mutations in Dlx3-HD are also able to cause TDO syndrome [6,18,19,22]. The substitutions of Q178 by Arg (Q178R mutant) [6,19] and of S182 by Phe (S182F mutant) [18] are causative mutations for TDO. A structural study revealed that residues Q178 (Q186 in Dlx5) and S182 (S190 in Dlx5) in the α3 helix of Dlx3-HD interacted with the T4 and T5 bases in the consensus DNA (Figure 1) [21]. Our study found that the T4 imino resonance was significantly down-field shifted by complex formation with Dlx3-HD (Figure 2C). In addition, the neighboring N179 and R180 resonances showed larger chemical shift changes upon binding to dlxDNA (Figure 4). It has also been reported that the R133P mutation in the L1 loop region of Dlx3-HD results in TDO syndrome [18]. According to the structural study [21], R133 formed H-bonding interactions with the T1 base of DNA (Figure 1). Data from a 1D imino NMR showed that the T1 imino resonance completely disappeared upon binding to Dlx3-HD (Figure 2C), indicating destabilization of the T1·A1’ base-pair. The CSP data showed that the I135 and Y136 amide signal located near R133 disappeared upon binding to DNA (Figure 4A). These data meant that residues R133, Q178, and S182 play important roles in target DNA recognition, which might be associated with TDO syndrome.

Salt concentration can have a dramatic effect on protein functions such as enzymatic catalysis, ligand binding, and DNA binding [23,24,25,26,27]. Our study found that Dlx3-HD has a strong binding affinity to consensus DNA duplex at [NaCl] = 250 mM (Figure 4), although the increment of [NaCl] from 100 to 250 mM leads to significant chemical shift changes in the amide resonances of free Dlx3-HD (Figure 3). This increment of [NaCl] made the Dlx3-HD-DNA interaction more dynamic, which was confirmed by fast exchange behavior in both the 1D imino proton spectra of DNA (Figure 2) and the 2D HSQC spectra of Dlx3-HD (Figure 4). However, when [NaCl] increased up to 500 mM, the Dlx3-HD exhibited much larger chemical shift changes in all amide signals (Figure 3), and its DNA binding affinity dramatically reduced (Figure 2 and Figure 4). These results suggest that Dlx3-HD exhibits a remarkable DNA binding affinity at cellular salt conditions (130–270 mM [28]) and that the dynamics of the Dlx3-HD–DNA interaction are modulated by varying salt concentrations.

## 4. Materials and Methods

### 4.1. Sample Preparations

The DNA oligomers d(CGTAATTGCC) and d(GGCAATTACG) were purchased from M-biotech Inc. (the Korean branch of IDT Inc.), purified by reverse-phase HPLC, and desalted using a Sephadex G-25 gel filtration column. The DNA duplex was prepared by dissolving two DNA strands at a 1:1 stoichiometric ratio in a 90% H_2_O/10% D_2_O NMR buffer containing 10 mM sodium phosphate (pH 6.0) and 100 mM NaCl. To produce ^15^N-labeled Dlx3-HD, BL21(DE3) bacteria expressing Dlx3-HD were grown in M9 medium containing 1 g/L of ^15^NH_4_Cl. The expressed proteins were purified by Ni–NTA affinity, followed by Sephacryl S-100 gel filtration chromatography (GE Healthcare, Chicago, IL, USA) on a GE AKTA FPLC. For NMR experiments, the purified proteins were concentrated to 1 mM in a 90% H_2_O/10% D_2_O buffer containing 10 mM sodium phosphate (pH 6.0) and 100 mM NaCl. The DNA and protein samples were dissolved in a 90% H_2_O/10% D_2_O NMR buffer containing 10 mM sodium phosphate (pH 6.0) and 100 mM NaCl.

### 4.2. NMR Experiments

All NMR experiments were performed on an Agilent DD2 700 NMR MHz spectrometer (GNU, Jinju, Korea) or a Bruker Avance-III 900-MHz NMR spectrometer (KBSI, Ochang, Korea) equipped with a triple resonance cryogenic probe. All ^1^H/^15^N HSQC and imino proton spectra were obtained for complexes prepared via the addition of DNA to 1 mM ^15^N-labeled Dlx3-HD or addition of ^15^N-labeled Dlx3-HD to 0.2 mM DNA. 1D NMR data were processed with the program M-Nova (Mestrelab, Santiago de Compostela, Spain), whereas 2D data were processed with the program NMRPipe [29] and analyzed with the program Sparky [30]. External 2-2-dimethyl-2-silapentane-5-sulfonate was used for the ^1^H and ^15^N references.

^1^H and ^15^N backbone resonance assignments for Dlx3-HD were obtained from the 3D NMR experiments, NOESY-^1^H/^15^N-HSQC and TOCSY-^1^H/^15^N-HSQC. The average chemical shift differences (Δδ_avg_) of the amide proton and nitrogen resonances between free Dlx3-HD and Dlx3-HD in complex with DNA were calculated using Equation (1):

(1) $$\Delta \delta_{\mathrm{avg}}=\sqrt{{\left(\Delta \delta_{H}\right)}^{2}+{\left(\frac{\Delta \delta_{N}}{5.88}\right)}^{2}}$$

where Δδ_H_ and Δδ_N_ are the chemical shift differences of the amide proton and nitrogen resonances, respectively.

## 5. Conclusions

Distal-less 3 (Dlx3) is a homeobox-containing transcription factor and plays a crucial role in the development and differentiation process. The homeobox domain of human Dlx3 (Dlx3-HD) selectively binds to the consensus site (5′-TAATT-3′) of the DNA duplex. This study found that the DNA binding of Dlx3-HD caused significant chemical shift changes in most of the amide resonances in the α3 helix and the disappearance of several amide signals in the L1 loop (Figure 4). We also found severe line-broadening of T1 imino resonance and chemical shift changes in the T4 imino resonance of dlxDNA caused by Dlx3-HD binding (Figure 2). These finding might be associated with TDO syndrome, which could be the result of R133P, Q178R, or S182F mutations in Dlx3-HD. This study revealed that the DNA binding affinity of Dlx3-HD is maintained at cellular salt concentrations, but that its dynamic properties can be modulated by varying salt concentrations. This unique structural and dynamic feature of Dlx3-HD plays an important role in target DNA recognition, which might be associated with TDO syndrome.

## Data Availability

The data presented in this study are available on request from the corresponding author.
